# Primary chronic osteomyelitis of the jaw: Rapid improvement after hormonal suppression in a girl with precocious puberty

**DOI:** 10.1016/j.bonr.2021.101089

**Published:** 2021-05-07

**Authors:** Caroline Robertsson, Lars Sävendahl, Carina Cardemil

**Affiliations:** a Unit of Cranio- & Maxillofacial Surgery, Karolinska University Hospital, Stockholm, Sweden; b Unit of Pediatric Endocrinology, Karolinska University Hospital, Stockholm, Sweden; c Department of Women's and Children's Health, Karolinska Institutet, Stockholm, Sweden; d Department of Molecular Medicine and Surgery, Karolinska Institutet, Stockholm, Sweden; e Department of Biomaterials, Institute of Clinical Sciences, Sahlgrenska Academy at University of Gothenburg, Gothenburg, Sweden

**Keywords:** CT, computed tomography, PCO, primary chronic osteomyelitis, CRMO, chronic recurrent multifocal osteomyelitis, LH, luteinizing hormone, FSH, follicle stimulating hormone, Primary chronic osteomyelitis, Chronic nonbacterial osteomyelitis, Mandible, Precocious puberty, Gonadotropin-releasing hormone

## Abstract

Primary chronic osteomyelitis (PCO) of the jaw is a non-infectious, inflammatory state of the jawbone of unknown etiology. In recurrent periods, these patients often exhibit swelling of the cheek, impaired ability to open their mouth as well as pain. Available treatments today include anti-inflammatory or antiresorptive drugs, hyperbaric oxygen, surgical decortication or resection followed by reconstruction where none of them have been described to lead to restored anatomy and complete relief of symptoms. We here report the unexpected complete regression of all clinical symptoms of PCO within three months after initiating pubertal suppression therapy with a gonadotropin-releasing hormone analogue in a 9-year-old girl with PCO of the jaw and early onset of pubertal development. Radiology of the jawbone confirmed complete PCO remission when performed 18 months after starting the hormone suppression therapy. To our knowledge, total regression of PCO in such a short period of time has not been described earlier suggesting an effect of the anti-hormonal therapy *per se*. In this case report, we discuss possible underlying mechanisms and hypothesize that anti-hormonal treatment could be a potential effective treatment in patients with PCO of the jaw.

## 1 Introduction

Osteomyelitis is an inflammatory condition of the bone and bone marrow which may be triggered by bacteria, previous surgical intervention or other trauma to the bone. There are many different types of osteomyelitis which differ in prevalence, symptoms, etiology and progression of the disease. Osteomyelitis can affect any bone in the body and be uni- or multifocal. The condition is in some cases associated with syndromes such as the Synovitis–acne–pustulosis–hyperostosis–osteitis Syndrome (SAPHO) or Majeeds syndrome (Stern and Ferguson, 2013). There are various classifications of osteomyelitis of the jaw where the literature describes both primary and secondary osteomyelitis. In some publications, the classification is based on the course of events at the onset of the disease, not on the cause itself (Wannfors, 1990). According to The Zurich classification system described by Baltensperger in 2008, primary chronic osteomyelitis lacks an external cause as opposed to the secondary form which is believed to be caused by external factors such as bacterial contamination through dental infection, wounds, trauma and other infections. Osteomyelitis of the jaw is presented as an acute condition or, if manifesting over a period of more than four weeks the osteomyelitis is considered to be chronic (Baltensperger and Eyrich, 2010).

Primary chronic osteomyelitis (PCO) of the jaw is a rare condition presenting as a non-infectious inflammatory state of the jawbone where patients often exhibit recurrent local swelling of the cheek, trismus as well as pain. Furthermore, radiology often shows increased bone formation and histology reveals a large number of osteoblasts and areas of immature bone.

The PCO diagnosis is based on a combined assessment of clinical, radiological and histopathological findings. As the clinical features can be difficult to interpret, a significant delay in diagnosing PCO often occurs in affected children or adolescents. Primary chronic osteomyelitis of the jaw causes long-term suffering from pain, swelling of the face and trismus. When left to have its natural course, it can in the long-term result in facial asymmetry. Today's treatment alternatives consist of anti-inflammatory medication (NSAID or steroids) (Baltensperger and Eyrich, 2010; Monsour and Dalton, 2010a), bisphosphonates (Gaal et al., 2020; van de Meent et al., 2020), hyperbaric oxygen treatment (Lentrodt et al., 2007), conservative treatment with occlusal splints, counselling/physiotherapy (van de Meent et al., 2019) and surgical decortication or resection (Theologie-Lygidakis et al., 2011; Bolognesi et al., 2020). Gaal et al. described a total of 22 patients treated for pediatric chronic nonbacterial osteomyelitis of the mandible and concluded that both anti-TNF and bisphosphonate treatments were superior to NSAIDs alone (11). Furthermore, a faster response to bisphosphonates was observed when compared to patients given anti-TNF treatment (11). To this date, published data on PCO consists of case reports, case series, small sample studies (Baltensperger and Eyrich, 2010; Monsour and Dalton, 2010a; van de Meent et al., 2019; Theologie-Lygidakis et al., 2011; Julien Saint Amand et al., 2017; Bevin et al., 2008; Baltensperger et al., 2004; Berglund et al., 2015; Eyrich et al., 2003; van de Meent et al., 2017) and reviews of the literature (van de Meent et al., 2020; Timme et al., 2020; Matharu et al., 2020). None of the mentioned treatments have been evaluated in prospective controlled trials. However, there are case reports of patients showing spontaneous improvement or a decrease of symptoms (Baltensperger and Eyrich, 2010; Jacobsson and Hollender, 1980; Jacobsson, 1984). To our knowledge, there are no descriptions of a rapid spontaneous complete remission of PCO when evaluating clinical signs, symptoms and three-dimensional radiography.

In this case report, we share our experience of a girl showing complete regression of PCO of the jaw soon after initiating hormone suppression treatment for central precocious puberty.

## 2 Case report

**Fig. 1:**
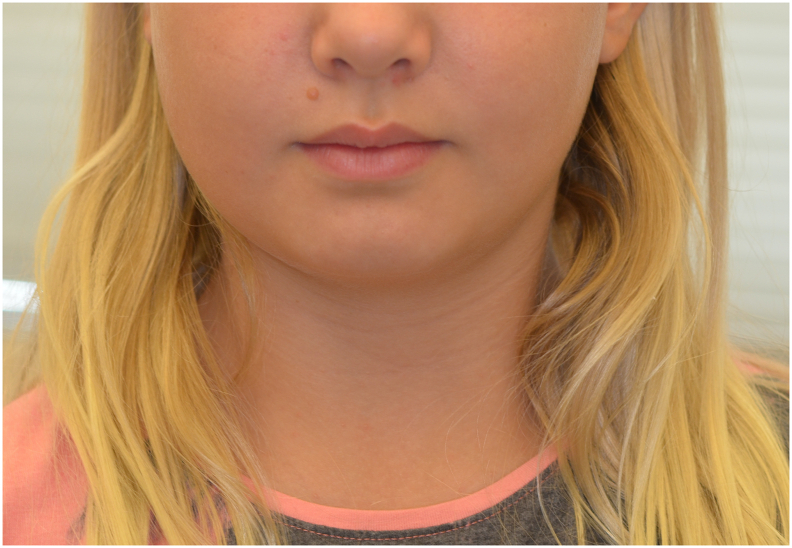
Mild swelling of the right cheek.

**Fig. 2:**
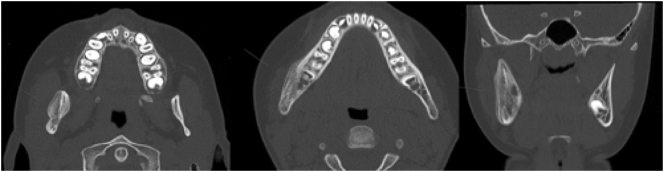
Bone apposition on the right body and ramus of the mandible as well as sclerosis combined with areas of osteolysis and periosteal thickening.

We present a case of a nine-year-old girl who experienced intermittent swelling of her right cheek associated with pain. After being evaluated by her family doctor, dentist, and a private ear, nose and throat (ENT) specialist, she was referred to the ENT Department at the Karolinska University Hospital where clinical examination ruled out parotid gland pathology and laboratory tests did not reveal any specific findings. A CT scan showed signs of osteomyelitis in her right mandible which rendered a referral to the Unit of Cranio- & Maxillofacial Surgery at Karolinska University Hospital where a mild swelling of the right cheek, trismus and a dull pain in the same area was found (Fig. 1). She described two- to three-week-long exacerbations of swelling and pain during the previous year, that recurred every two or three months. Radiology demonstrated increased mandible width, periosteal thickening and no clear difference between cortical and cancellous bone (Fig. 2). Altogether, these findings lead to the decision to obtain a bone biopsy and bacterial culture from the right mandibular body. The biopsy was retrieved under general anesthesia using an intraoral approach with as much isolation as possible to avoid contamination. Adequate isolation was verified by the absence of bacterial pathogens within the bone.

**Fig. 3:**
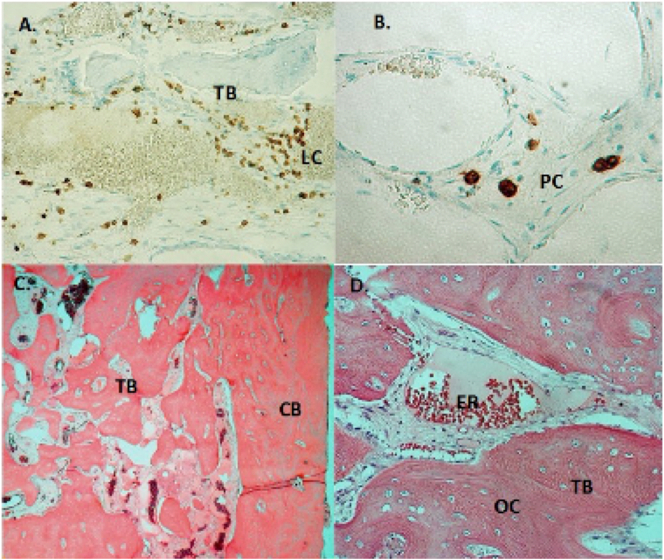
A. Light micrograph of decalcified trabecular bone (TB) and marrow spaces from the right angle of the mandible. Staining with CD45 (LCA/leucocyte common antigen), showed accumulation of leukocytes (LC) in a highly vascularized fibrous stroma indicative of chronic osteomyelitis. Original magnification 250×. B. Light micrograph of trabecular bone and marrow spaces from the right angle of the mandible. Immunohistochemistry for CD138, showed presence of plasma cells (PC) in a fibrous stroma indicative of chronic osteomyelitis. Original magnification 400×. C. Light micrograph of decalcified cortical (CB) and sclerotic trabecular bone (TB) surrounding marrow spaces with a highly vascularized fibrous stroma without signs of necrosis or bone sequestration. Staining with Hematoxylin eosin, original magnification 100×. D. Light micrograph of bone from the right angle of the mandible stained with Hematoxylin eosin showing immature trabecular bone (TB) with irregularly distributed osteocytes (OC) indicative of rapid bone formation. Dilated blood vessel with erythrocytes (ER) can be seen in the fibrous stroma of the marrow spaces. Original magnification 250×.

Histopathological examination showed an appearance of the mandibular bone in accordance with primary chronic osteomyelitis. Culture of harvested bone revealed no presence of bacterial agents (Fig. 3).

As depicted in Fig. 6, the patient was prescribed ibuprofen at the time of diagnosis at the Unit of Cranio- & Maxillofacial Surgery for symptom release which had a moderate effect. She was instructed to take this regularly (200 mg 3 times daily) for at least 2 months. Then instructed to take it whenever needed.

**Fig. 4:**
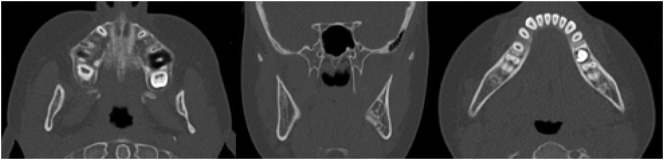
CT scan performed 18 months after starting triptorelin treatment showed complete restoration of the jaw anatomy and total regression of the osteomyelitis.

Parallel to developing jaw pathology, pubertal signs including breast development and growth acceleration were reported at 8 years of age. This led to a referral to the Pediatric Endocrinology Clinic at the Astrid Lindgren Children's Hospital where clinical evaluation confirmed Tanner stage 3 breast development, growth acceleration, two years accelerated bone age, and pubertal levels of estradiol, luteinizing hormone (LH) and follicle stimulating hormone (FSH). Altogether confirming that the patient had entered puberty at a relatively young age. As requested by the patient and her parents, pubertal suppression treatment was initiated at 9 years of age by administering monthly intramuscular injections of an agonist analogue of gonadotropin-releasing hormone, triptorelin (3.75 mg), a treatment well known to reversibly suppress the hypothalamic-pituitary-gonadal axis. At clinical follow-up six months after starting triptorelin injections, the patient reported that all symptoms of PCO including pain and swelling of the jaw had disappeared within two-three months after initiating the treatment. At follow-up visits 11 and 18 months after initiating the triptorelin treatment, no new periods of swelling or pain from the jaw were reported and no clinical signs of jaw inflammation were noted. Radiology performed 18 months after starting the triptorelin treatment confirmed complete remission of the osteomyelitis (Fig. 4).

**Fig. 5:**
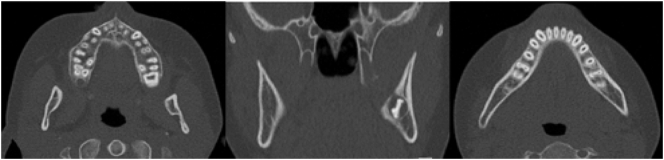
CT scan six months after the last injection of triptorelin showed completely restored anatomy and a total regress of the osteomyelitis in the right mandible.

**Fig. 6:**
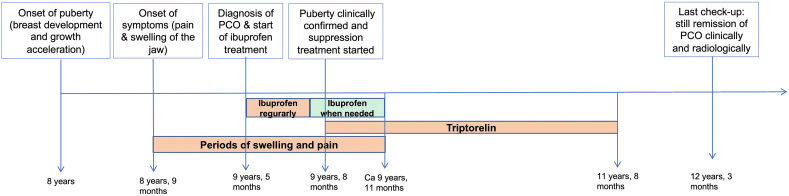
Timeline illustrating the series of events.

After 24 months of pubertal suppression, the triptorelin injections were stopped according to the initial treatment plan. Clinical check-up six months after the last injection with triptorelin revealed no sign of recurrence of the PCO, neither clinically nor radiologically, and sporadic menstrual periods were reported starting approximately four months after the last injection. Hence, one can assume that her serum estradiol had increased to normal pubertal levels six months after the last injection of triptorelin while she showed no signs of recurrence of PCO (Fig. 5). Fig. 6 illustrates the series of events.

## 3 Discussion

We present a case of a nine-year-old girl showing rapid improvement of PCO of the jaw after initiating anti-hormonal treatment to suppress early pubertal development. To our knowledge, such a prompt resolution of this condition has not been described earlier.

In the recent decade, it has been questioned whether PCO is mainly caused by bacterial infection and a new theory has evolved ruling out the component of bacteria in the development of the condition. Hence, studies have been directed mostly at confirming or excluding bacterial agents (Baltensperger and Eyrich, 2010). The disease of PCO is not fully understood and is often misdiagnosed as osteomyelitis caused by a dental infection or other more common causes. Therefore, the diagnosis and treatment of PCO are often delayed (Camison et al., 2018). The diffuse onset of symptoms in PCO contributes to the difficulties in setting the diagnosis in this patient group (Baltensperger and Eyrich, 2010). This is well reflected in the timeline of the case described in this report where the diagnosis was set nine months after the debut of symptoms and the patient suffered from moderate swelling, trismus and pain varying in intensity over the course of time. These findings are all consistent with symptoms associated with PCO as described in the literature. The patient's radiological appearance is also congruous with lesions described in PCO of the jaw (Baltensperger and Eyrich, 2010; Bevin et al., 2008; Berglund et al., 2015; Eyrich et al., 2003).

Since this is such an uncommon disease, not many patients with co-existing morbidities have been studied and even fewer have been investigated regarding their pubertal development. PCO may present at any age but many cases described in the literature are of growing individuals, a time period when hormonal levels often peak (Stern and Ferguson, 2013; Baltensperger and Eyrich, 2010; Monsour and Dalton, 2010a; Gaal et al., 2020; Bevin et al., 2008; Baltensperger et al., 2004; Berglund et al., 2015; Eyrich et al., 2003; Agarwal et al., 2014; Obel et al., 2013; Taddio et al., 2017; Monsour and Dalton, 2010b; van Merkesteyn et al., 1990). Gaal et al. presented 22 cases of PCO where lytic and sclerotic lesions of the mandible as well as an increased bone width were described (Gaal et al., 2020). This concurs well with findings in the case described here, showing comparable radiological appearance in the right mandibular body at the time of diagnosis. In 1975, Jacobsson et al. described an interesting patient with fibro-osseous dysplasia mimicking diffuse sclerosing osteomyelitis of the mandible who had recurrent periods of exacerbations corresponding to her menstrual periods that could be managed by progesterone treatment (Jacobsson et al., 1975). Although fibro-osseous dysplasia and osteomyelitis is not the same disease, sex hormones may contribute to the development of both these bone pathologies. To our knowledge, a possible hormonal influence on the development of PCO has not been discussed in the literature so far. Several other reports have confirmed a female predominance and typical presentation during puberty (Baltensperger and Eyrich, 2010; Baltensperger et al., 2004; Berglund et al., 2015; Frid et al., 2009). Døving et al. described a case of a healthy female who during the first trimester of her pregnancy developed PCO of the jaw (Døving et al., 2019). In the early months of pregnancy, there is a spike in estradiol levels, a finding similar to what is observed in pubertal girls. These few but important observations raise the question if sex hormones play a role in the development of PCO.

In bone, constant remodeling takes place allowing not only growth and healing to occur but also to maintain a homeostasis between calcium and phosphate. Remodeling is a process dependent on the balance between the bone-resorbing osteoclasts and the bone-forming osteoblasts. The interaction between these cells is regulated by many systemic and local factors and an imbalance in this interaction can cause a variety of pathological bone conditions. Sex hormone receptors are expressed in all tissues and estrogen has a direct effect on osteoblast and osteoclast activity (Khosla et al., 2012). Estrogens and androgens have a strong impact on skeletal growth and are also involved in skeletal homeostasis. The sex hormone receptors have different roles in trabecular and cortical bone remodeling, and periosteal and endosteal surfaces of the bone also respond in different ways to changes in growth factors, hormones and mechanical stress (Manolagas et al., 2013). Estrogens protect against bone loss by, among other things, impeding the development of new osteoclasts. Thus, sex hormones have a very significant impact on skeletal homeostasis throughout life and the knowledge of the mechanisms behind these processes is constantly increasing.

Triptorelin is a gonadotropin-releasing hormone analogue that is used to treat central precocious puberty in girls before the age of eight and in boys before the age of 10, as well as hormone dependent advanced prostate cancer, hormone dependent breast cancer in premenopausal women and endometriosis. The mechanism of action of triptorelin is to prevent the activation of the hypothalamic-pituitary-gonadal axis (HPG), which in turn results in cessation of production of LH/FSH from the pituitary gland. This subsequently results in a pronounced reduction in estrogen production and an inhibition of the development of the reproductive organs. Known side effects of triptorelin include osteoporosis and decreased bone mineralization, depression, back pain, decreased libido, bone paresthesia, hyperhidrosis and asthenia (Furman, 2016). As estrogens have direct effects on bone metabolism (Iravani et al., 2017), a drastic increase in estrogen levels, as seen during puberty or pregnancy (Døving et al., 2019), or a pronounced suppression of estrogen levels, as seen during anti-hormonal treatment, could have the potential to modulate conditions of bone pathology, such as PCO. Several *in vivo* studies have shown that bone growth is affected by gonadotropin-releasing hormone analogues (Iravani et al., 2017; Pasquino et al., 2008; Magiakou et al., 2010). However, it is not the direct effect of a gonadotropin-releasing hormone analogue that affects bone metabolism, but the effect it has on the pituitary gland to stop producing LH and FSH, which is then followed by a deprivation of estrogen that systemically affects the skeleton (Furman, 2016).

The estrogen deprivation that follows the reduced production of LH and FSH caused by the drug, has a direct effect on the osteoclasts (Manolagas et al., 2013). As estrogens also inhibit several inflammatory cytokines, the decline in estrogen levels increases the levels of pro-inflammatory cytokines (Pacifici, 1996; Lerner, 2006). These pro-inflammatory cytokines activate the osteoclasts and stimulate osteoclastogenesis resulting in increased bone resorption and osteoporosis (Sambrook and Cooper, 2006). Loss of estrogens also affects osteoblast progenitor cells through decreased estrogen receptor-alpha expression resulting in a lower response to mechanical stimulation of the bone, thus to less bone formation (Manolagas et al., 2013; Iravani et al., 2017). Treating girls with central precocious puberty with triptorelin is a clinical routine which has been shown to not impair adult height, skeletal mineralization or future reproduction (Pasquino et al., 2008). The patient described in this case report entered puberty during her eighth year, and she was both physically and mentally ahead of her peers. This resulted in the possibility of slowing down her entrance into adolescence and she started the treatment at nine years of age. In the case described here, there were no reported side effects of the triptorelin treatment other than the patient reporting some discomfort having the monthly injections. In addition, she showed a complete recovery from her symptoms of PCO at the time when she was severely estrogen deprived. At clinical follow-up six months after ending triptorelin treatment, there were no signs of side effects of the treatment and her menstrual periods had commenced. Nor were there any observations suggesting recurrence of PCO. Rapid regression of all symptoms of PCO with complete normalization of bone morphology in terms of thickness, cortical and cancellous appearance has rarely been described in the literature. Nevertheless, the biological events leading to the fast healing in our PCO case could be coincidental. It would therefore be of great interest to further investigate the effect of modulation of hormonal levels in patients with PCO. If there is a hormonal influence on the development of PCO remains to be established and there is a need to clarify the mechanism of action behind this rare condition. Another group of patients that could be included in further studies are cases with Chronic Recurrent Multifocal Osteomyelitis (CRMO) where several bones of the body are affected without a known bacterial or traumatic incident in the patient history (Navallas et al., 2020). As with PCO, the etiology is also here not fully understood and different treatments are suggested, however there are no treatment guidelines (Timme et al., 2020; Taddio et al., 2017).

## 4 Conclusion

We present a case of a pubertal girl where complete regression of PCO of the jaw was observed soon after starting treatment with a gonadotropin-releasing hormone analogue. The rapid improvement suggests a possible effect of the anti-hormonal treatment *per se*. Experimental studies and clinical trials are needed to establish if there is a hormonal component involved in the development of PCO and if hormonal repression could improve or resolve the condition.

## Funding

This research did not receive any specific grant from funding agencies in the public, commercial, or not-for-profit sectors.

## Author contributions

CR, LS and CC designed the study. CR and LS treated the patient and collected data. CR, LS and CC analyzed the data. CR, LS and CC wrote the manuscript.

## Ethical approval

This project was approved by the Swedish Ethical Review Authority and the patient and parents described in this article have signed informed consent of publication of this data.

## Declaration of competing interest

None.
